# Quantifying Dynamic Changes in Plantar Pressure Gradient in Diabetics with Peripheral Neuropathy

**DOI:** 10.3389/fbioe.2016.00054

**Published:** 2016-07-19

**Authors:** Chi-Wen Lung, Elizabeth T. Hsiao-Wecksler, Stephanie Burns, Fang Lin, Yih-Kuen Jan

**Affiliations:** ^1^Rehabilitation Engineering Laboratory, Department of Kinesiology and Community Health, University of Illinois at Urbana-Champaign, Champaign, IL, USA; ^2^Department of Creative Product Design, Asia University, Taichung, Taiwan; ^3^Department of Mechanical Science and Engineering, University of Illinois at Urbana-Champaign, Urbana, IL, USA; ^4^Department of Physical Therapy, Langston University, Langston, OK, USA; ^5^Center for Lower Extremity Ambulatory Research, Rosalind Franklin University, North Chicago, IL, USA

**Keywords:** diabetes, plantar pressure, pressure gradient, diabetic foot ulcers, peripheral neuropathy

## Abstract

Diabetic foot ulcers remain one of the most serious complications of diabetes. Peak plantar pressure (PPP) and peak pressure gradient (PPG) during walking have been shown to be associated with the development of diabetic foot ulcers. To gain further insight into the mechanical etiology of diabetic foot ulcers, examination of the pressure gradient angle (PGA) has been recently proposed. The PGA quantifies directional variation or orientation of the pressure gradient during walking and provides a measure of whether pressure gradient patterns are concentrated or dispersed along the plantar surface. We hypothesized that diabetics at risk of foot ulceration would have smaller PGA in key plantar regions, suggesting less movement of the pressure gradient over time. A total of 27 participants were studied, including 19 diabetics with peripheral neuropathy and 8 non-diabetic control subjects. A foot pressure measurement system was used to measure plantar pressures during walking. PPP, PPG, and PGA were calculated for four foot regions – first toe (T1), first metatarsal head (M1), second metatarsal head (M2), and heel (HL). Consistent with prior studies, PPP and PPG were significantly larger in the diabetic group compared with non-diabetic controls in the T1 and M1 regions, but not M2 or HL. For example, PPP was 165% (*P* = 0.02) and PPG was 214% (*P* < 0.001) larger in T1. PGA was found to be significantly smaller in the diabetic group in T1 (46%, *P* = 0.04), suggesting a more concentrated pressure gradient pattern under the toe. The proposed PGA may improve our understanding of the role of pressure gradient on the risk of diabetic foot ulcers.

## Introduction

Diabetic foot ulcers remain one of the most serious complications of diabetes mellitus (Burns and Jan, 2012). It is estimated that 15% of diabetics will develop a foot ulcer during their lifetime, and 2–3% of the population may develop a foot ulcer annually (Burns and Jan, 2012). In 2000–2001, the cost of treating a diabetic foot ulcer averaged $13,179 per episode, which increased with severity level (Stockl et al., 2004). In 2007, the total cost of treatment was $58 billion in the United States (American Diabetes Association, 2008). Diabetic peripheral neuropathy causes not only loss of protective sensation but also changes in the soft tissues of the foot as well as dryness of the skin that can lead to excessive formation of callus (Burns and Jan, 2012; Jan et al., 2013a,b). These changes affect ambulatory function that may lead to high plantar pressures in diabetics (Lung and Jan, 2012; Jan et al., 2013a). The repetitive high pressure insults to the plantar surface of the diabetic foot have been shown to be associated with the development of foot ulcers (Veves et al., 1992; Bus, 2012; Patry et al., 2013).

To better understand the influence of abnormal plantar pressure distributions on the development of diabetic foot ulcers, peak plantar pressure (PPP) has been widely used to assess trauma to the soft tissues of the diabetic foot (Veves et al., 1992; Pitei et al., 1999b; Caselli et al., 2002). However, the threshold of the PPP for causing diabetic foot ulcers remains largely unknown (Armstrong et al., 1998a; Mak et al., 2010). Furthermore, only a moderate correlation between the location of diabetic foot ulcers and the PPP has been reported (Veves et al., 1992). Lavery et al. (2003) suggested that the PPP alone is not adequate to predict the development of skin breakdown; they suggested that other variables and methods should be investigated to predict the risk of diabetic foot ulcers. Because of the complex geometry and non-linear material properties of the foot, the forces, pressures, and stresses acting on the plantar soft tissues exhibit a complex behavior (Gefen et al., 2000; Chen et al., 2010; Jan et al., 2013a).

Mueller et al. (2005) introduced another index, peak pressure gradient (PPG), for characterizing the largest change in plantar pressure between adjacent pressure sensors pixels of a pressure mapping system. Thus, the PPG is a metric that captures the largest pressure gradient observed in a given region of the plantar surface. The PPG has shown promise in predicting the development of diabetic foot ulcers in many previous studies (Mueller et al., 2005, 2008; Zou et al., 2007; Lott et al., 2008), and various ethnic people (Lung et al., 2013; Fawzy et al., 2014). Supriadi et al. (2014) further defined a cutoff value of PPG for the risk threshold of pressure ulcers.

According to the principle of PPG, a low average PPP coupled with high PPG is more damaging than a high PPG on its own. Lott et al. (2008) demonstrated a significant relationship among PPP, PPG, and maximal shear stress in the diabetic foot. The high PPG may contribute to skin breakdown because high PPG may cause large shear stresses within the plantar soft tissues (Mueller et al., 2005). Jan et al. (2013a) further demonstrated that changes in viscoelastic properties of plantar soft tissues contribute to abnormal PPP and PPG patterns in diabetics with peripheral neuropathy. Due to the complex dynamics experienced by the ankle and foot during gait and the structural and functional changes associated with diabetes, the current definition of PPG may not be adequate to identify diabetics at risk of diabetic foot ulcers (Jan et al., 2013a).

According to the definition of PPG proposed by Mueller et al. (2005), the PPG is calculated based on PPP distributions during the overall contact time without consideration of time-varying features of PPP locations during the gait cycle. However, the directions of consecutive maximal pressure gradients may vary during gait. Therefore, PPG by itself does not account for variations in pressure gradient direction during the stance phase of the gait cycle, and varying pressure gradient direction may cause a more complicated deformation on the underlying plantar soft tissues. To account for the mean directional variations of the pressure gradient during the stance phase, a new metric pressure gradient angle (PGA) was defined in our previous study (Lung et al., 2013). The PGA quantifies the time-varying directions of instantaneous PPG.

**Figure 1:**
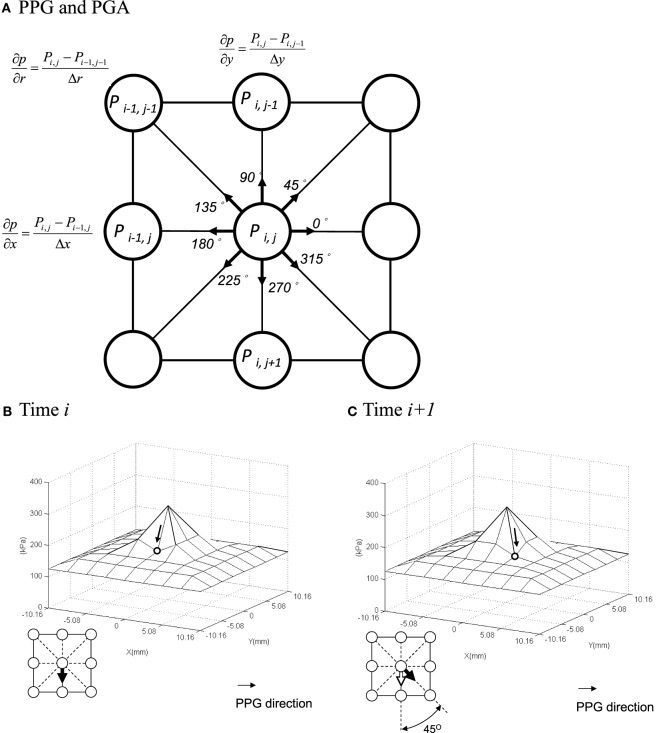
**Illustrations of the peak pressure gradient (PPG) and pressure gradient angle (PGA)**. **(A)** Calculation of the PPG. **(B,C)** Quantify the time-varying directional variations of pressure gradients (i.e., PGA). The PGA is 45° from **(B)** time *i* to **(C)** time *i* + 1. Although the PPP and PPG values are the same, the directions of PGA are different. The arrow represents the direction of peak pressure gradient.

Theoretically, the PGA increases with the dispersion of pressure (Figure 1B) and decreases with the concentration of pressure (Figure 1C). As shown previously, we demonstrated that the PGA provides additional information to quantify the pressure gradient patterns (Lung et al., 2013). The PGA was found to be significantly smaller for diabetics compared with controls under the first toe, thus suggesting a greater concentration of pressure gradient in persons with diabetes. The purpose of the current study was to further quantify the differences between diabetics and healthy controls during walking in different plantar regions at high risk of ulceration. We hypothesized that values of PPP and PPG would be greater and the PGA would be lower in diabetics compared with non-diabetics. Our long-term goal is to further improve our understanding of the role of plantar pressures on the pathogenesis of diabetic foot ulcers.

## Materials and Methods

### Participants

Twenty-seven volunteers were recruited, including 19 type 2 diabetics (10 males) and 8 non-diabetic healthy controls (4 males). Subjects with gross foot deformities (except minor toe clawing) and prior foot amputations/major surgeries were excluded for a more homogeneous population. The demographic data of the control group were age 23.1 ± 3.2 years, weight 66.8 ± 21.3 kg, height 1.66 ± 0.12 m, body mass index (BMI) 24.0 ± 6.9 kg/m^2^, heart rate 69.1 ± 7.8 beats/min, systolic blood pressure 108.3 ± 12.7 mmHg, and diastolic blood pressure 68.6 ± 8.7 mmHg. The demographic data of the diabetic group were age 42.2 ± 12.6 years, weight 94.0 ± 21.7 kg, height 1.74 ± 0.15 m, BMI 31.4 ± 7.6 kg/m^2^, heart rate 76.1 ± 13.5 beats/min, systolic blood pressure 129.3 ± 21.7 mmHg, and diastolic blood pressure 79.2 ± 14.7 mmHg. The fasting blood glucose level and duration of diabetes were 137.6 ± 10.7 mg/dL and 9.2 ± 2.3 years, respectively. All diabetics had plantar foot ulceration in the past and had healed more than 3 months at the time of the experiment; they also had peripheral neuropathy confirmed by the inability to sense a 5.07 Semmes–Weinstein monofilament in at least four locations of the plantar foot (Apelqvist et al., 2000). This study was approved by an institutional review board for human subject research. The research protocol was explained to the volunteers who signed an informed consent form.

### Plantar Pressure Measurements

The F-scan system (Tekscan, South Boston, MA, USA) was used to collect plantar pressure data of the right foot during walking at a self-selected pace (ranged from 2 to 4 km/hr) in standardized shoes (Mueller and Strube, 1996; Pitei et al., 1999b). Each F-scan in-shoe sensor contains 960 sensing pixels (sensels). The size of each pixel is 5.08 mm × 5.08 mm. The sensor was placed between the subject’s sock and the insole of the shoe. All subjects wore ambulatory shoes with a 1′ heel (Altrex, Teaneck, NJ, USA). The right shoe was worn with its standard insert and a thin cotton sock. Subjects wore the sensor inside the right shoe for 3–5 min of walking before calibration (Mueller and Strube, 1996; Pitei et al., 1999b). The sensor was then calibrated according to the manufacturer’s guidelines and was consistent with use reported in the literature (Mueller and Strube, 1996; Pitei et al., 1999b). Data were collected at 200 Hz during two walking trials in the same direction on a 15-m walkway immediately after calibration (Mueller and Strube, 1996; Pitei et al., 1999b).

### Data Analysis and Statistics

**Figure 2:**
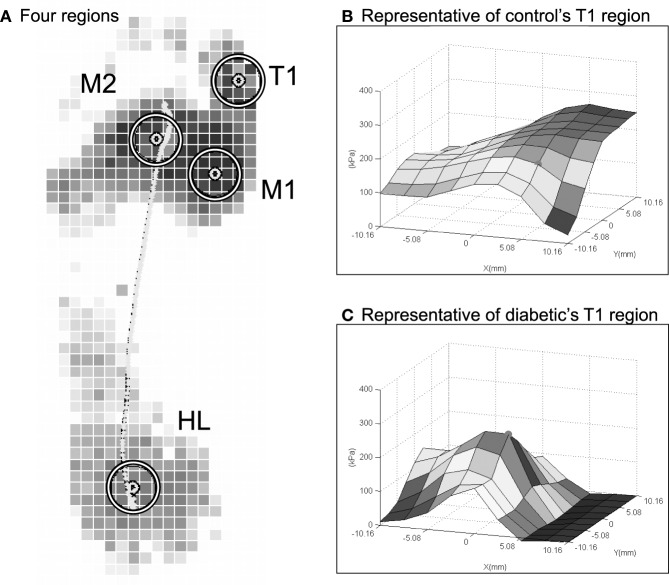
**Examples of PPP angle at the first toe in a representative control and a representative diabetic**. **(A)** Four plantar regions are defined. **(B)** Control: PPP = 262 kPa, PPG = 29 kPa/mm, and PGA = 135°. **(C)** Diabetic: PPP = 228 kPa, PPG = 58 kPa/mm, and PGA = 0°. For these two sample cases in **(B,C)**, the PPP between the control and diabetic are similar, but the PPG and PGA are quite different.

Data from the three middle steps were processed to calculate the average PPP, PPG, and PGA across the three steps. These average values were determined in four plantar regions at high risk of diabetic foot ulcers (Armstrong et al., 1998a; Lung and Jan, 2012). The four regions were the first toe (T1), the first metatarsal head (M1), the second metatarsal head (M2), and the heel (HL) (Figure 2A) (Armstrong et al., 1998a; Lung and Jan, 2012).

The PPP was defined as (Mueller et al., 2005; Zou et al., 2007):

(1) PPP= max (p)

where *p* is the pressure distribution within each of the four plantar region surfaces.

After the maximal value of the *p* sensor was identified, eight sensors around the *p* sensor were chosen. A composite area by these nine sensors was a defined area for the further analyses of all plantar pressure-related variables. According to the convention of Mueller et al., the PPG was determined in a defined area [a 3 × 3 box of sensing pixels on the F-scan sensor (232.3 mm^2^)] around a central node (Figure 1A). Node positions were generated using a bicubic polynomial spline function (Mueller et al., 2005). The PPG was calculated by determining the greatest difference in pressure from one node (half sensel apart) to the next according to row, column, and diagonal directions. Thus, each node has eight gradient vectors $\vec{r}$ but has one peak vector at the same time, max$\left(\vec{r}\right)$ (Figure 1A). The PPG is the magnitude of the largest peak gradient vector for a given gait cycle. The PPG can be calculated as (Mueller et al., 2005; Zou et al., 2007):

(2) $$\text{PPG magnitude }=\text{ max}\left(\frac{\partial p}{\partial r}|{{\;}_{\left(x_{p},y_{p}\right)}}_{\;}\right)$$

where (∂*p*/∂*r*)|(*x_p_, y_p_*) (space rate of change of pressure on the plantar surface) is the directional derivative of pressure *p* at the node for a given plantar region (*x_p_, y_p_*) on the plantar surface in any of the eight directions given by the vector $\vec{r}$.

The PGA can be determined by considering the directional variations of the peak gradient vector between two consecutive frames of the time-varying, instantaneous PPG, i.e., max$\left({\vec{r}}_{i}\right)$ and max$\left({\vec{r}}_{i+1}\right)$ (Figure 1A). The angle α can be computed from the dot product of the magnitudes of these two vectors. The PGA is the average change in α during a stance phase of gait cycle (Lung et al., 2013):

(3) $$\text{PGA}=\frac{1}{N-1}\sum_{i=1}^{N-1}\left(\alpha_{i+1}-\alpha_{i}\right)$$

where α is the angle of the pressure gradient vector at time *i*, and *N* is the time when the instantaneous PPP is more than half of the overall PPP. As shown in our previous study (Lung et al., 2013), the results of PGA were stable when the PGA was calculated by the instantaneous PPP of more than 50% sensors. The selection of pressures with more than half PPP is to exclude unstable PGA associated with small plantar pressures.

We analyzed the intra-observer variability by using intraclass correlation coefficients (ICC). The distribution patterns of all variables were analyzed using the one-sample Kolmogorov–Smirnov test. The differences in the PPP, PPG, and PGA between diabetics and controls were examined using the Student’s *t*-test (Klaesner et al., 2002). The values were presented as the mean ± SD. Correlations between the PPP, PPG, and PGA were determined using a Pearson product-moment correlation analysis (Mueller et al., 2005). The level of the significance was set at 0.05 (Rothman, 1990; Perneger, 1998).

## Results

Correlation coefficients among all measurements of intra-observer were high enough (ICC = 0.79). All results of this study were normally distributed. Examples of PPP and PPG at the first toe in a diabetic and a healthy control are provided to illustrate the concept of the PGA in Figure 2. Despite that the PPP values of the control and the diabetic subjects were similar (~250 kPa), PPP distributions for controls (Figure 2A) were spatially flatter than diabetic PPP distributions (Figure 2B). These distributions lead to the PPG as 29 kPa/mm in control and 58 kPa/mm in diabetics, respectively. In this case, the PGA was larger at 45° in a healthy control than 0° in a diabetic.

**Figure 3:**
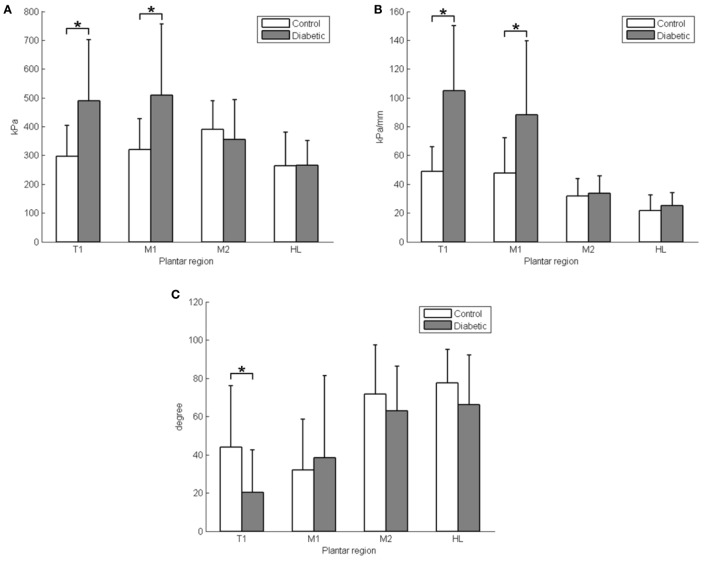
**The comparison of PPP, PPG, and PGA between the diabetic and control groups**. **(A)** Peak plantar pressure (PPP); **(B)** peak pressure gradient (PPG); **(C)** pressure gradient angle (PGA). T1, first toe; M1, first metatarsal head; M2, second metatarsal head; and HL, heel; *P < 0.05, values are means with SDs.

A detailed comparison of differences between the diabetic and control groups and the four plantar areas are provided in Table 1. The PPP value at the first toe was significantly smaller in the control group (297.0 ± 107.8 kPa) compared with the diabetic group (489.4 ± 211.4 kPa, *P* < 0.05, Figure 3A). The PPP value at the first metatarsal head was significantly smaller in the control group (319.9 ± 107.8 kPa) compared with the diabetic group (509.6 ± 245.8 kPa, *P* < 0.05, Figure 3A). The PPG value at the first toe was significantly smaller in the control group (49.0 ± 16.8 kPa/mm) compared with the diabetic group (104.9 ± 45.3 kPa/mm, *P* < 0.05, Figure 3B). The PPG value at the first metatarsal head was significantly smaller in the control group (47.7 ± 24.4 kPa/mm) compared with the diabetic group (88.3 ± 51.2 kPa/mm, *P* < 0.05, Figure 3B). The PGA at the first toe was significantly greater in the control group (44.0° ± 32.2°) compared with the diabetic group (20.4° ± 22.0°, *P* < 0.05, Figure 3C). No significant differences were found for the other two areas, second metatarsal head and heel.

**Table 1:** **Correlations among variables in the control and diabetic groups**.

	Control	Diabetic
PPP and PPG	0.58*	0.86*
PPP and PGA	−0.12	−0.44*
PPG and PGA	−0.59*	−0.59*
PPP and age	0.05	0.08
PPG and age	−0.06	0.11
PGA and age	−0.02	−0.07
PPP and weight	−0.34	0.05
PPG and weight	−0.34	−0.08
PGA and weight	−0.13	−0.11
PPP and BMI	−0.34	0.04
PPG and BMI	−0.26	−0.02
PGA and BMI	−0.16	−0.16

*PPP, peak plantar pressure (kPa); PPG, peak pressure gradient (kPa/mm); PGA, plantar pressure gradient angle (degree)*.

*Significant correlations P < 0.05.

**Figure 4:**
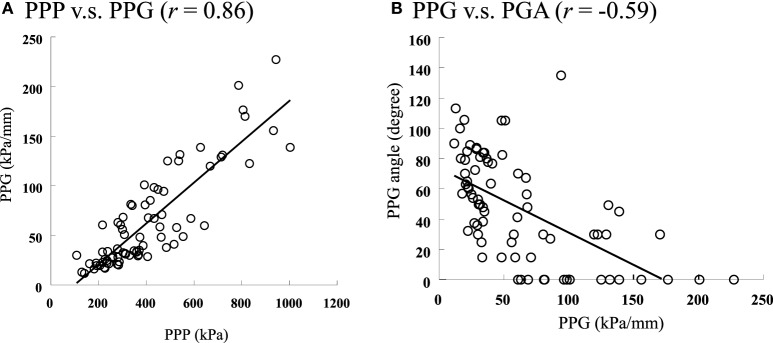
**The scatter plots to show the relationships among the PPP, PPG, and PGA in diabetics**. **(A)** The relationship between PPP and PPG, PPP vs. PPG (*r* = 0.86). **(B)** The relationship between PPG and PGA, PPG vs. PGA (*r* = −0.59).

The correlations between the PPP, PPG, and PGA are listed in Table 1. The correlation between the PPP and PPG was *r* = 0.58 in the control group (*P* < 0.05) and *r* = 0.86 in the diabetic group (*P* < 0.05, Figure 4A). The correlation between PPG and PGA was *r* = −0.59 in the control group (*P* < 0.05) and *r* = −0.59 in the diabetic group (*P* < 0.05, Figure 4B). The correlation between PPP and PGA was significant for the diabetic group (*r* = −0.44, *P* < 0.05).

## Discussion

The pathogenesis of diabetic foot ulcers is a multifactorial process that depends on complex interactions between the internal structure and viability of the foot and external forces and pressures during walking (Yarnitzky et al., 2006; Atlas et al., 2009; Lung and Jan, 2012; Jan et al., 2013a). The internal structure of the foot includes geometric shape and alignment of hard and soft tissues, tissue mechanical properties, and individual tissue tolerance to loading. The external forces include the dynamic patterns of plantar pressures that can be characterized by various analyses (e.g., PPP, PPG, and PGA). We believe the PPG may be a more sensitive indicator of injury risk than the PPP. The spatial change in pressure across the surface of the skin appears to be an important component of predicting these subsurface shear stresses. We previously defined the peak pressure gradient (PPG) as the greatest spatial change in plantar pressure around the PPP location (Fernando et al., 1991).

The location of PPP is identified, and then the spatial change in plantar pressure is determined for every direction around the PPP location. The greatest spatial change in plantar pressure (i.e., the greatest slope of the pressure distribution) is the PPG. Based on mechanical theories (Sackfield et al., 2013), we believe pressures that change substantially across the surface of skin (i.e., high PPG) are more damaging than high pressures distributed equally across the skin surface. For example, the hydrostatic pressures experienced by the skin of deep-sea divers may be very high, but divers do not experience skin breakdown because these high pressures are distributed evenly across the surface of the skin (i.e., they have a very low PPG). The relationship among various magnitudes of PPG and the resultant calculated PMSS is illustrated in Figure 1 using methods previously reported.

In our previous study (Jan et al., 2013a), abnormal PPP and PPG were related to alterations in viscoelastic properties of plantar soft tissues of the diabetic foot. Furthermore, diabetics with peripheral neuropathy, who have loss of sensation, may significantly change their gait patterns. Such abnormal gait patterns inevitably alter plantar pressure distributions in diabetics. The methods and findings of this study aimed to contribute to the understanding of the pathogenesis of diabetic foot ulcers, and our findings alone will not adequate to address the risk of diabetic foot ulcers.

The results support our hypotheses that the PPP and PPG of the diabetic group were significantly higher than in the control group at the first toe and first metatarsal head, and the PGA in the diabetic group were significantly lower than in the control group at the first toe. The PGA shows a significant correlation with the PPG in the diabetic group. The proposed new variable, PGA, was able to further define the pressure gradient patterns in diabetics and may provide additional insight into the mechanism of the influences of PPG on the development of diabetic foot ulcers.

Peak plantar pressure values were significantly greater in the diabetic group than in the control group at the first toe and first metatarsal head. The mean PPP were greater at the first toe and first metatarsal head of the diabetic group than in the control group in this study. However, in the heel, no significant increases were observed in diabetics. These results are consistent with the literature. Perry et al. (2002) showed that the highest plantar pressure occurred at the first metatarsal head in diabetics. They proposed that the diabetes-associated stiffening of the plantar soft tissues at the pad of the first toe and first metatarsal head may cause this abnormal PPP. The epidermal layer of plantar soft tissues was also reported to become stiffer in diabetics (Chao et al., 2011). Gefen et al. (2001) found that the stiffness of the soft tissues of the first metatarsal head was substantially larger than other plantar regions in diabetics. Zheng et al. (2000b) also demonstrated that the Young’s modulus (elasticity) of plantar tissues of diabetics increased at different plantar areas; the maximum increase (160%) was observed in the area at the first metatarsal head, and the second maximum increase was at the first toe, while no significant increase was observed in the heel area. Our results support that the first toe and first metatarsal head are at higher risk for foot ulceration during walking (Gefen, 2003).

The mean PPG were 214% greater in the first toe and 185% in the first metatarsal head in the diabetic group than in the control group. The increase in PPG at the first toe and first metatarsal head areas in diabetics may be attributed to a significant limitation of motion at the metatarsophalangeal joints. The exact pathogenesis of the limited joint mobility in diabetics remains unclear but is thought to be related to the high stiffness of quasi-linear viscoelasticity in the soft tissues (Lung and Jan, 2012; Jan et al., 2013a) and the progressive stiffening of the collagen-containing tissues due to accumulation of advanced glycation end products (AGEs) (Burns and Jan, 2012). The diabetic foot with limited motion at the metatarsophalangeal joints significantly reduces shock absorbing ability and may cause an abnormal plantar pressure distribution (Zimny et al., 2004; D’Ambrogi et al., 2005). As illustrated in Figure 2, the PPP alone may not be able to fully describe the risk of diabetic foot ulcers, and the PPG may provide additional useful information. Fernando et al. (1991) showed that limited joint mobility may be a major factor in causing abnormally high PPP and contributes to foot ulceration. The authors also demonstrated that abnormal plantar foot pressures alone did not predict the location of foot ulcers. Furthermore, Fawzy et al. (2014) indicated that the forefoot PPG in diabetics with neuropathy was ~1.5 times higher than that of diabetics without neuropathy. The PPG has previously been reported as related to the posture changes (Hobson, 1992) and materials of insoles change (Kang and Mak, 1997) in previous studies. The range of motion of the first metatarsophalangeal joint significantly reduced (D’Ambrogi et al., 2005), which may play an important role of the increased PPG in first toe and first metatarsal head. Our results of the PPP and PPG in the diabetic and control groups support the principle of assessing both the PPP and PPG to predict diabetic foot ulcers.

A noted result in this study was that the PPP and PPG of diabetics were not significantly different from controls in the heel region and second metatarsal head. The reason may be explained by the trajectory of the center of pressure. Because shear stress is highly correlated with the PPG (Zou et al., 2007), the trajectory of the center of pressure passes through the heel region and second metatarsal head during walking (Mann et al., 1988). This interpretation is consistent with the results reported by Armstrong et al. (1998b). They reported that only 1 and 6% of the wounds occurred in the heel region and second metatarsal head. Lord and Hosein (2000) also reported that these two regions had lower shear stresses during walking.

The mean PGA was greater at the first toe in the diabetic group than in the control group in this study. As hypothesized, the PGA was significantly lower in diabetics as compared with controls. Ahmed et al. (2010) reported that the most common sites of diabetic foot ulcers were in the plantar surface of the first toe. About one-third of diabetics develop a callus at the first toe. Plantar callus is associated with high vertical and shear forces in diabetics (Pitei et al., 1999a). When the callus is removed, plantar pressures are reduced by 32.1% in diabetics (Pitei et al., 1999a). This finding indicates that a callus may act as a foreign body elevating plantar pressures. As high shear stresses are associated with foot ulcers (Manorama et al., 2010), the low PGA in diabetics may be negatively related with shear stresses. Further studies need to establish the relationship between PGA and shear stresses. Calluses are generally not harmful but may sometimes lead to changes in PGA that may aggravate the risk of foot ulceration (Lung and Jan, 2012; Jan et al., 2013a). To investigate the relationship between calluses and PGA, future research can classify the type of influence of PGA on the formation of calluses in response.

We postulate that the PGA described in this study can be integrated into current risk assessments of diabetic foot ulcers. The PGA has the potential to improve the design of orthotic devices in the prevention of diabetic foot ulcers. The correlation between PPG and PPP was considerably higher in the diabetic group than in the control group (*r* = 0.86 vs. 0.58). As Mueller et al. (2005) defined, the PPG represents the spatial changes in the pressure in the region of the PPP. From a mechanical standpoint, a sharp change in the highest pressures, i.e., a high PPG, may lead to internal stress concentrations and shearing of soft tissues, causing soft tissue injury (Zhang et al., 1994; Manorama et al., 2010). Although the underlying cause of the increased PPG remains unclear, the involvement of PGA has been implicated. As high PPG appears to have a negative correlation of PGA (*r* = −0.59), it is recommended that further research needs to test and evaluate potential interventions to increase the PGA in diabetics for preventing diabetic foot ulcers. For example, medications may be used to alter the AGEs accumulation for the reduction of stiffness of plantar soft tissues in diabetics, and thereby possibly reversing the changes in PGA and reducing risk for foot ulcers. In addition, orthotic devices may be designed and constructed to compensate for the changes that cause higher PPP and PPG and lower PGA.

This study is a first step in comprehensively investigating the importance of the PGA as an indicator of diabetic foot ulcers. A benefit of using this approach to estimate PGA is that only the pressure distribution is needed for data entry. However, PGA requires several assumptions. One of the assumptions is the plantar soft tissue is assumed to be isotropic, homogeneous, and linearly elastic (Jan et al., 2013a). The assumption of small strain deformation is violated because plantar soft tissue deformation can be up to 35–46% (De Clercq et al., 1994; Cavanagh, 1999). PGA was found to be significantly smaller in the diabetic group in T1. The soft tissue depth may affect the values of PGA. Zheng et al. (2000a) showed that the tissue thickness of the T1 is thinner than M1, M2, and HL. PGA may thus play a significant role in thinner soft tissue of diabetes foot.

There are several limitations of this study. First, the sample size was small, which might impede the power of the statistical analysis. We did not have an age and BMI matched control. Factors, such as age and BMI, may affect our results. We performed a correlation analysis to examine whether demographic data (age, body weight, and BMI) significantly contributed to the results observed in this study. We did not find any significant correlation between the demographic data and variables of this study. Although the diabetic group was 27 kg heavier than the non-diabetic group, obesity and being overweight are usually observed in the type 2 diabetics and may inherently contribute to abnormal plantar pressure distributions (Qatanani and Lazar, 2007; Atlas et al., 2009). Cavanagh et al. (1991) demonstrated that there was lack of a strong relationship between body weight and plantar pressure parameters. We did not perform a longitudinal follow-up of the incidence of diabetic foot ulcer to examine the power of using the PGA on predicting foot ulcers. Whether changes in PGA are associated with a higher risk for foot ulcers require additional investigation.

## Conclusion

We introduced PGA to further quantify the pressure gradient patterns in diabetics in this study and successfully demonstrated that diabetics have higher PPP and PPG, and lower PGA especially at the first toe compared with non-diabetics. Our method and findings may contribute to the understanding of the role of plantar pressures in the development of diabetic foot ulcers. Our findings provide a basis to further explore the time-varying features of plantar pressure distributions associated with the functional and structural changes of the diabetic foot.

## Author Contributions

Study concept and design: Y-KJ. Acquisition of data: C-WL. Analysis and interpretation of data: C-WL, EH-W, SB, FL, and Y-KJ. Drafting of the manuscript: C-WL and Y-KJ. Critical revision of manuscript for important intellectual content: C-WL, EH-W, SB, FL, and Y-KJ. Obtained funding: Y-KJ.

## Conflict of Interest Statement

The authors declare that the research was conducted in the absence of any commercial or financial relationships that could be construed as a potential conflict of interest.
